# The Interplay of Biomarkers and Psychosocial Variables in IPV Perpetration

**DOI:** 10.3390/bs15081075

**Published:** 2025-08-07

**Authors:** Arthur Cantos, María L. Mondolfi, K. Daniel O’Leary

**Affiliations:** 1 Department of Psychological Science, The University of Texas Rio Grande Valley, Edinburg, TX 78539, USA; 2 Associated Centre Pontevedra, National Distance Education University, 36162 Pontevedra, Spain; marmondolfi@pontevedra.uned.es; 3 Department of Psychology, Stony Brook University, Stony Brook, NY 11794, USA; daniel.oleary@stonybrook.edu

**Keywords:** intimate partner violence, testosterone, cortisol, adverse childhood experiences, emotion regulation

## Abstract

Relevant biopsychosocial factors, including testosterone (T) and cortisol (C) levels, adverse childhood experiences (ACEs), and difficulties in emotion regulation, have been implicated in IPV perpetration. However, further research is needed to clarify how biomarkers and psychosocial variables interact. The authors herein predicted that emotion regulation difficulties would moderate the association between ACES and IPV perpetration. The sample consisted of 30 IPV perpetrators aged 18 to 51 (*M* = 30.80, *SD* = 8.43) and 30 control non-perpetrator participants aged 18–35 (*M* = 24.13; *SD* = 4.28). All participants provided saliva samples to assess T and C levels and completed a sociodemographic questionnaire that included questions related to ACEs, and the Difficulties in Emotion Regulation Scale (DERS). Higher levels of T and T/C, greater difficulties in emotion regulation, and higher prevalence of ACEs were found to significantly differentiate the IPV perpetrators from the non-IPV perpetrators. However, difficulties in emotional regulation did not emerge as a significant moderator between these variables, and only one of four subscales of the DERS, emotional awareness, was significantly associated with both testosterone and IPV. These results are in accord with meta-analytic results which found that DERS scores are higher in IPV perpetrators than non-perpetrators but that there was a very small association between emotional dysregulation and IPV (0.14), and emotional awareness was not associated with IPV. Similarly, effect sizes for ER variables are strong as differentiators of perpetrators and non-perpetrators.. Given the relative strength of psychological variables as moderators of childhood trauma and IPV, anger would appear to be a much stronger moderator as it is a much stronger predictor of IPV than emotional dysregulation.

## 1. Introduction

Intimate partner violence (IPV) is defined by the World Health Organization (WHO, 2024) as any act in the context of an intimate relationship that causes physical, sexual, or psychological harm. IPV has become a major public health concern. According to the last National Intimate Partner and Sexual Violence Survey, the lifetime prevalence of IPV victimization in the United States is 47.3% for women and 44.2% for men (Leemis et al., 2022). The existing literature highlights the insidious effects of IPV, including physical injuries, deteriorating general health, depression, anxiety, posttraumatic stress disorder, substance abuse, dissatisfaction with the relationship, and heightened suicidal risk (Caldwell et al., 2012; Chu et al., 2023; Rasmussen et al., 2023). Although the majority of the IPV literature focuses on the effects on female victims, male IPV victimization has been described as having similar psychological consequences (Scott-Storey et al., 2023). However, a higher percentage (50%) of all women than men (10%) killed are murdered by their partners (AbiNader et al., 2023). Furthermore, recent research underscores the often-bidirectional occurrence of IPV and its broader impact on family systems and communities (Alexander & Johnson, 2023; Keilholtz et al., 2023). Evidence-based research is therefore necessary to inform policies and interventions.

According to J. C. Babcock et al. (2004) two main approaches underlie the interventions of the court-mandated treatment programs for IPV perpetrators. First is the Duluth model which is based on feminist theory and sociological concepts and is not a psychotherapeutic intervention but a psychoeducational perspective. Second is the cognitive behavioral therapy (CBT) approach, a psychotherapeutic intervention orientated to modifying violent behaviors and learning alternatives to the use of violence. Meta-analytic studies (J. C. Babcock et al., 2004; Feder & Wilson, 2005) revealed both approaches having small overall effects and, thus, a minimal impact on reducing recidivism that is not much more significant than the effect of being arrested. More recently, Travers et al. (2021) conducted a meta-analytic analysis that corroborated these findings, underscoring the limited efficacy of traditional interventions based on Duluth’s model and CBT. However, Travers et al. (2021) also identified the risk-need-responsibility (RNR) framework as a promising alternative, with significant medium effect sizes on recidivism in research studies with up to two-year follow-ups. The RNR framework consists of adapting treatments to individual forensic risk assessments, considering factors like antisocial traits, family and marital circumstances, socioeconomic conditions, and substance abuse. The need to individualize interventions based on the characteristics of the perpetrators and the type of violence perpetrated has also been pointed out by proponents of one size does not fit all (Cantos & O’Leary, 2014; J. Babcock et al., 2016). Cantos et al. (2019), in a review of 456 IPV perpetrators on probation, found that participation in the Duluth type intervention predicted both recidivism and time to recidivism for the Generally Violent but not Family Only perpetrators who participated in treatment. Specifically, GV men were responsive to treatment whereas FO men were not. However, both groups had significant reductions in IPV, but the family only offenders may have responded to the arrest and desire to maintain partner or family ties independent of treatment. Further evidence that perpetrators with different profiles would benefit from different types of interventions comes from a study by Redondo et al. (2019) in which following completion of a cognitive-behavioral therapy program, there was a significant reduction in intimate partner violence for both subtypes of partner violent men with anger problems, under controlled and overcontrolled, although the under controlled individuals continued to present more frequent psychological aggression and dominant and jealous tactics. Recidivism was predicted by pretreatment levels of hostility and treatment completion.

In summary, the complexity and implications of IPV compel multicausal approaches to understanding and addressing it. In this vein, Raine (2002) found that high levels of testosterone (T), impulsivity, and aggressive behaviors in children can lead to early rejection and a further heightened risk of delinquency and therefore stressed the importance of considering biosocial factors in the study of aggressive behaviors to understand how biological markers can interact with environments and mutually influence each other.

Likewise, from an ecological perspective, O’Leary et al. (2007) found that dominance and jealousy-based dyadic dynamics in couples, along with other factors like a lack of social support, negative life experiences, and individual psychopathology, could predict IPV perpetration in both men and women. Thus, considering bio/psychosocial variables when designing interventions and treatments for IPV perpetrators may enhance their efficacy and reduce recidivism. Both adverse childhood experiences and emotion dysregulation are two promising variables to consider.

As a metatheoretical framework for understanding and predicting a range of behaviors, the I cubed (I^3^) model (Finkel & Eckhardt, 2013; Finkel, 2014) has been useful for researchers in developing testable hypotheses and understanding aggressive behaviors. The I^3^ model focuses on three processes that are considered to be orthogonal—meaning any of them can be high or low independently of the others. These three processes are as follows. (I) Instigation: This refers to exposure to environmental cues or targets that normatively lead to likely behaviors (e.g., being insulted may instigate aggressive responses). (II) Impellance: This includes dispositional or situational factors that increase the urge to respond with a specific behavior when exposed to a target object (e.g., trait impulsivity intensifying aggressive responses to provocation). (III) Inhibition: This encompasses both situational and stable factors that increase the likelihood of suppressing the enactment of the behavior despite instigation and impellance (e.g., high self-control preventing aggression). Building on this framework, the “perfect storm” theory posits that the highest likelihood of enacting a behavior (especially problematic ones, like aggression) occurs when instigation and impellance are strong and inhibition is weak.

### 1.1. Adverse Childhood Experiences (ACEs), Emotion Dysregulation, and IPV

Gratz and Roemer (2004) proposed a definition of emotion regulation to obtain construct validity for the Difficulties in Emotion Regulation Scale (DERS), one of the most used instruments in this field. Their definition of this ability encompasses the main conceptual and empirical approaches in the area and involves the following aspects:

(a) awareness and understanding of emotions; (b) acceptance of emotions; (c) ability to control impulsive behaviors and behave in accordance with desired goals when experiencing negative emotions; and (d) ability to use situationally appropriate emotion regulation strategies flexibly to modulate emotional responses as desired in order to meet individual goals and situational demands (Gratz & Roemer, 2004, p. 42).

Thus, a deficit in any of the aspects mentioned in the proposed definition is considered emotion dysregulation or difficulties in emotion regulation, according to Gratz and Roemer (2004). Over the last two decades, there has been a surge in research on emotion dysregulation, with a focus on its role in everyday experiences and associations with trauma and psychopathology (MacNamara et al., 2023). Previous research has shown that difficulties in emotion regulation are positively associated with physical, sexual, and psychological IPV perpetration (Maloney et al., 2023).

Adverse childhood experiences (ACEs) have been described as stressful events that can disrupt children’s development and jeopardize further mental health, as cohort studies have demonstrated. Physical, emotional or sexual abuse, parental divorce, dysfunctional households, witnessing violence or abuse, and residing in noxious environments are examples of ACEs (Yoon et al., 2023). These kinds of trauma trajectories have been analyzed as risk factors for aggressive behaviors, yielding a positive association with early onset of offenses and arrests and a cumulative effect consisting of each additional ACE increasing the risk for recidivism and more violent offenses (Baglivio et al., 2015). Similarly, Stinson et al. (2021) found that the prevalence of ACEs was significantly larger in a sample of adult psychiatric forensic inpatients than in a community sample. In this context, some previous studies, such as that by Machisa et al. (2016), described the direct effect of ACEs on male IPV perpetration as mediated by poor mental health, particularly posttraumatic stress disorder (PTSD).

The intergenerational transmission of violence model is based on social learning theory, which states that imitation and modeling are the means of transmission for violent patterns through generations. Studies under this model have linked family of origin violence (FOV) to IPV perpetration and victimization. Consequently, not only being abused as a child but also witnessing IPV between parents can be risk factors for both IPV perpetration and victimization in adulthood (Ehrensaft & Langhinrichsen-Rohling, 2022). Within this framework, childhood physical abuse has been linked to IPV perpetration and victimization (Black et al., 2010; Puno et al., 2023). Likewise, Islam et al. (2017) found that witnessing father-to-mother IPV was linked to increased odds of endorsing attitudes that justify spouse battering and perpetrating IPV. Moreover, in a study by Manchikanti-Gómez (2011), child abuse and dating violence in adolescence were found to be associated with IPV incidence in adulthood, with victimization more prevalent in women and perpetration in men.

A study by Orozco-Vargas et al. (2021) showed that having witnessed IPV was indirectly related to IPV via emotion dysregulation, and the correlations between these variables were stronger in male participants than in females. Likewise, Oliveros and Coleman (2019) found that having witnessed IPV perpetrated by either parent was linked to emotion regulation issues, IPV perpetration, and victimization in male participants, while for women, these associations were only significant in the case of IPV having been perpetrated by their mothers. The study also found that child abuse was connected to IPV perpetration, with emotion regulation difficulties playing a mediating role. For men, this mediating effect was only significant if they had been abused by their fathers, but for women, it was significant if they had either been abused by their mothers or fathers.

Similarly, Iverson et al. (2014) found anger-related dysregulation to mediate the relationship between witnessing IPV in childhood and IPV victimization in early adulthood for both men and women. More recently, Meddeb et al. (2023), in a study with a forensic sample of adult perpetrators, found ACEs to be associated with aggressive and antisocial behaviors and difficulties in emotion regulation, including impulse control, acceptance of one’s emotions, and the ability to maintain goal-oriented behaviors and access emotional regulation strategies during stressful situations. Difficulties in goal-oriented behaviors mediated the relationship between ACEs and aggression, while lack of emotional awareness predicted more callous aggression in that same study. A systematic review by Zhang et al. (2023) determined early exposure to IPV to negatively affect the development of self-regulatory strategies, including emotional and behavioral regulation, and recommended continuing research on the mechanisms implied. Therefore, the need for research focusing on underscoring the mechanistic factors linking ACEs and IPV in adulthood prevails in this field.

### 1.2. The Role of Testosterone and Cortisol on Aggressiveness and IPV

A review conducted by Archer (2006) to evaluate the role of T on dominance and aggression arising from comparative psychology concluded that higher levels of T are weakly but positively associated with dominance tendencies and aggressive responses to threatening or competitive situations. Nevertheless, according to Archer (2006), such responses can also be regulated by individuals’ experiences and successful reactions to challenges in their lives.

Regarding IPV perpetrated by men, Cohan et al. (2003) found that dominance and aggression are not solely associated with men’s T levels but also influenced by their female partners’ T levels. Likewise, Kaiser and Powers (2006) found that the female partner’s T levels can act as a moderating variable in this context. Thus, T levels and aggression in men appear to be regulated by environmental factors and the dynamics of the dyadic relationship, particularly in cases of IPV.

Further studies on testosterone and behavior have followed the dual-hormone hypothesis, which is based on the relationship between testosterone (T) and cortisol (C) and their effects on brain structures. Studies with humans and other mammals have found that T induces activity in the striatal dopaminergic system (the reward system), while C has the opposite effect, so it has been associated with the threat system. But the relationship between T and C is complex and they are also related to anger stimuli responses. Further, it has been demonstrated that T administration activates amygdala reactivity to angry faces and C levels have the opposite effect, so the increase in C leads attention away from the stimulus (Denson et al., 2013).

Apparently, the neuroendocrine systems regulated by T and C oppositely regulate aggressive responses, with C modulating the effects of T on aggressive behaviors (Montoya et al., 2012; Terburg et al., 2009). Elevated levels of T are linked to dominance-seeking behavior, while decreased levels of C are associated with avoidance behavior, so this combined hormonal profile might have a more pronounced effect on aggression than either hormone alone (Blair et al., 2004; Mehta & Prasad, 2015; Terburg et al., 2009). Relatedly, in a study by O’Connor et al. (2002), T did not show up as a significant predictive variable, but impulsivity was found to be significantly associated with aggressive behavior in a sample of community eugonadal men. Nonetheless, Coccaro et al. (2007) found a positive association between basal T levels and venturesomeness (sensation seeking) in personality-disordered men.

A pilot study by Brown et al. (2007) indicated that lower C levels and higher T levels were associated with increased levels of anger in men. Specifically, the association of higher T levels with anger expression was more consistent than the association (whether negative or positive) between C or dehydroepiandrosterone (DHEA) levels and anger expression. Moreover, C levels alone were less likely to correlate with impulsive aggressive behaviors compared to T levels. In a similar vein, T and sensation seeking were found to be associated in both male and female individuals without psychological disorders by Borraz-León et al. (2023). Likewise, a meta-analytic study by Kurat and Mata’s (2018) revealed a positive association between T, estradiol, impulsivity and risk-taking. Dekkers et al. (2019) also found a positive association between T and dominant behaviors, which was only significant in the presence of low C levels.

A literature review by Pinto et al. (2010) on IPV perpetration and biological markers highlighted that elevated levels of T, or reduced serotonin activity, might imply an alteration of neuronal function that could be linked to the proclivity to rapidly respond to situations as threatening in the contexts of IPV perpetration. A study on stress responses by Romero-Martínez et al. (2013a) found that IPV perpetrators exhibited higher testosterone-cortisol ratios (T/C) both during the preparatory phase and after a stress-induction task compared to control individuals. This suggests that IPV perpetrators may be more prone to engage in violent behaviors due to faster anticipatory responses and more intense reactions. Interestingly, higher T/C ratios were also linked to higher self-esteem and better mental health outcomes in perpetrators, while in controls it was associated with increased anger traits. In a similar study by Romero-Martínez et al. (2013b), higher basal T levels were found to be associated with increased levels of anger and anxiety following a stress-induction task in both male IPV perpetrators and controls. Of note, after the stressor, C levels were only elevated among control participants, suggesting a potential habituation effect or hypoactivity in the HPA axis in perpetrators, reducing their reactivity to psychological stress. Additionally, after stress induction, IPV perpetrators exhibited decreased T levels alongside worsened mood and anxiety. These changes were more pronounced in individuals with higher baseline T levels, which were also correlated with elevated anger levels. Romero-Martínez et al. (2015) found that although IPV perpetrators did not differ from controls in the T/C ratio, only in perpetrator was there a positive relationship between antisocial, borderline and narcissistic personality traits, anger expression and basal T/C ratio. The authors also stated that the T/C ratio plays a moderating role in the relationship of antisocial and borderline traits with anger expression. In IPV perpetrators the T/C ratio may explain why certain personality traits are associated with high risk of becoming violent. Romero-Martínez et al. (2015) also found that IPV perpetrators showed poorer emotion recognition and higher attention switching costs than controls although they did not differ in attention to detail and hormonal parameters (T/C ratio). An additional study, consistent with the dual-hormone hypothesis, found that (1) greater AUCg T/C ratios were associated with greater aggression and (2) T/C ratio–aggression associations were weaker under provoked conditions but did not differ as a function of sex or ADHD status (Manigault et al., 2019).

Consistent with accumulated empirical evidence, a previous study utilizing the same sample as the current one Cantos et al. (2024) identified T, C, and T/C levels as significant variables for distinguishing men placed on probation for IPV from those in a community sample of non-violent controls as well as predicting IPV severity among perpetrators. The study by Cantos et al. (2024) also revealed that IPV perpetrators had higher basal T levels and T/C ratios than non-perpetrators, and that higher levels of T were positively associated with more severe IPV aggression among perpetrators. In the same study, IPV perpetrators exhibited higher C levels after exposure to a stressor than non-perpetrators, and trait anger as was identified as a moderator variable between T and T/C levels and perpetration/non-perpetration.

Based on these prior findings, there is a growing interest in identifying psychological variables that may help explain how biological and environmental risk factors contribute to IPV perpetration. Due to its associations with both hormonal activity and ACEs, emotion regulation might provide a better understanding of the psychological mechanisms underlying IPV perpetration and reveal targeted strategies for prevention and support in at-risk populations. It is important to note that the literature reviewed is correlational and given there has been no manipulation of variables in these studies, causality cannot be established.

## 2. The Current Study

The existing literature underscores the importance of continuing to investigate variables associated with IPV perpetration to inform tailored treatments and enhance their efficacy. While T and C are significant biological markers linked to the likelihood of IPV perpetration, directly manipulating these hormones in treatment interventions raises ethical concerns and is not feasible. Nonetheless, studies have revealed significant associations between these biomarkers and psychological variables suitable for treatment interventions, including emotional reactions to stress that differentiate IPV perpetrators from non-perpetrators and predict more severe aggressions among perpetrators. In this context, we aim to identify additional psychological moderators that might shed light on the influence of these biological factors on IPV perpetration. Therefore, this study aims to determine the combined influence of biological and environmental variables in elucidating the risk of IPV perpetration. For these purposes, we analyze differences between IPV male perpetrators and non-perpetrator controls in emotion regulation difficulties and ACEs, as well as the links between emotion regulation and hormonal biomarkers (T and T/C).

Our hypotheses are as follows: (i) IPV perpetrators will exhibit higher scores in difficulties in emotion regulation, and report more incidence of ACEs than non-violent controls; (ii) difficulties in emotion regulation will positively correlate with T, T/C levels, and IPV perpetration; (iii) difficulties in emotion regulation will moderate the relationship between both T levels and T/C ratios and the likelihood of IPV perpetration; (iv) the conjunction of T levels and T/C ratios, as biological variables and ACEs (child abuse, witnessing IPV) as environmental variables, will influence the probability of IPV perpetration.

## 3. Materials and Methods

We employed a contrasted groups design consisting of an experimental group of men with a history of IPV perpetration and a control group of men with no such history.

### 3.1. Participants

The total sample comprised 60 adult male volunteers, divided into two groups: 30 who had perpetrated intimate partner violence and were placed on probation (research group) and 30 who had not (control group). The IPV perpetrator volunteers were recruited from the Hidalgo County Probation Department in Edinburg, Texas, situated in the Rio Grande Valley (RGV). Inclusion criteria for this group were: (a) being male and over the age of 18; (b) being on probation for an IPV-related offense in Hidalgo County; and (c) not taking any medication that could interfere with hormone measures at the time of participation.

Flyers were posted at neighborhood community centers to recruit participants for the control group, which comprised males over the age of 18 without any prior history of intimate partner violence (IPV) and not taking any medication that could affect hormone measures.

### 3.2. Measures

#### 3.2.1. The Difficulties in Emotion Regulation Scale (DERS)

The DERS is a self-report scale that asks respondents how often each item applies to their experiences, with responses ranging from 1 (almost never) to 5 (almost always). The scale comprises 36 items with high internal consistency (α = 0.94); six subscales have Cronbach’s alphas > 0.80 (Gratz & Roemer, 2004). The subscales reflect difficulties within the following dimensions: nonacceptance of one’s own emotional responses to stress (acceptance), difficulties to engage in goal-directed behavior when negative emotions are experienced (goals), inability to maintain self-control over behavior when experiencing negative emotions (impulse), lack of consciousness of or inattention to one’s own emotional responses (awareness), perceived inability to access emotional regulation strategies once upset (strategies), lack of emotional clarity regarding the nature of one’s feelings (clarity), and the total score in difficulties in emotion regulation strategies (DERS) when facing stressful situations or negative emotions. In this study, the total DERS and subscales showed reliability ranging from acceptable to excellent, as indicated by the following Cronbach’s α values: DERS α = 0.907, acceptance α = 0.843, goals α = 0.832, impulse α = 0.795, awareness α = 0.726, strategies α = 0.704, clarity α = 0.728.

#### 3.2.2. Testosterone and Cortisol Levels

Saliva samples were processed using a human testosterone and cortisol ELISA from Enzo Life Sciences (Farmingdale, NY, USA: Cat# ADI-900-176 and ADI-900-071). The assay sensitivity was 2.6 and 56.72 (picograms per milliliter) for T and C, respectively. Samples were measured in duplicate, and our analysis used the sample mean. The curve was a standard curve using known concentrations included in the kit of the two hormones, and the concentration of testosterone and cortisol was expressed as pg/mL. With intra- and inter-assay variation coefficients of less than 10%, good precision was attained.

#### 3.2.3. Demographics Questionnaire

Participants completed an inquiry about personal antecedents and socioeconomic information, including: age, incomes, education level, occupation, marital and familial status, having been hit by parents (mother, father, or both) as a child, having witnessed physical IPV between parents (unidirectional or bidirectional) as a child, having mental health problems and having received treatment for those in the past, alcohol and drug use, and antecedents of violent crimes or non-violent offenses (for the research group, different from the incident that made them put on probation).

#### 3.2.4. ACEs (Child Abuse, Witnessing IPV)

As part of the demographics questionnaire, participants were asked (1) whether they were hit by their mother, their father, or both. They were also asked (2) whether they witnessed physical IPV between parents (unidirectional or bidirectional) as a child.

### 3.3. Procedure

Participants provided informed consent before the research plan and its implications were thoroughly explained to them. All study protocols underwent review and approval by the Institutional Review Board of The University of Texas Rio Grande Valley Participants were schedule for an individual session in the morning to complete the research questionnaires and provide a saliva sample. Saliva samples for hormonal analysis were gathered at the beginning of the session, and questionnaires were administered afterwards. As reported in (Cantos et al. 2024) the following procedure was used to take the salivary assays: It was requested that participants avoid eating a major meal, foods with high sugar or acidity, high caffeine content, alcohol, nicotine, or drugs (prescription/over-the-counter-medication), brushing their teeth, or performing exercise two hours before arriving to their appointment. Participants were then asked to provide two saliva samples for hormonal analysis: Drool 1 was gathered at the beginning of the session and Drool 2 was gathered at the end of the session. Participants were asked what time they woke up the morning of session 1 and this time was recorded and used as a control variable in analyses to control the natural diurnal cycle of cortisol. The sampling of saliva was non-invasive; the participant was asked to slowly drool into a straw which was attached to a small plastic vial. Research assistants immediately secured the vial and placed it in a −20-degree Celsius freezer to be transferred to the university endocrinology research laboratory.

Research participants were evaluated at an assigned office at the Hidalgo County Community Supervision and Corrections Department, while control participants were interviewed at the university community psychology training clinic in a lab research office. Participants were instructed to avoid consuming a major meal, foods with high sugar or acidity, high caffeine content, alcohol, nicotine, or drugs (both prescription and over-the-counter medication), brushing their teeth, or engaging in exercise for two hours before the appointment. All participants were provided with a gift card for $25 following their participation.

### 3.4. Data Analysis

We calculated descriptive analyses and bivariate correlations between the variables in the study using Pearson and point biserial correlation coefficients. To check for significant differences between the research group (IPV perpetrators) and control group (non-perpetrators), *t*-tests with Levene’s test for equality of variances were calculated for the following variables: age, difficulties in emotion regulation strategies, T levels, and T/C ratios. Crosstabulation was calculated to determine differences between groups in terms of ACEs, including having witnessed IPV among parents and having experienced child abuse (having been hit by parents). To ascertain the effects of T levels, T/C ratios, difficulties in emotion regulation strategies, and ACEs, binary logistic regression models were estimated.

To investigate the effects of T, T/C, and impulsivity on IPV perpetration, moderated by difficulties in emotion regulation, multiple regressions were estimated. Two separate analyses were carried out: one using T as the predictor variable and the other using T/C ratios. The outcome variable for these analyses was categorized as IPV perpetration or non-perpetration (control or research group). These analyses involved entering age as a covariate on step one, the conditional main effects (T or T/C) on step 2, and the interaction between the predictor variables (T or T/C) and the moderating variables (difficulties in emotion regulation) on step 3. A level significance of 0.05 was used for all analyses. All the analyses were conducted using SPSS version 28.

To test our hypotheses, we used disjunction testing, within the multiple regression and moderation analyses. Disjunction testing is a statistical approach particularly useful in theory-driven research that anticipates several non-mutually exclusive predictors or interaction terms (e.g., biological, psychological, and environmental factors) contributing to the outcome. Disjunction testing provides more flexibility than standard conjunction-based approaches by not requiring all predictors to be significant to support a hypothesis. Given the multifactorial nature of IPV and the possibility of outcomes emerging through unique or overlapping mechanisms, this approach provided an appropriate framework for testing our model (Meehl, 1990; Abelson, 1995; Lakens, 2017).

Given the limited sample size, which reflects the challenges of recruiting from a hard-to-reach population, we conducted a post hoc power analysis using simulation methods (Lane, n.d.). This analysis was not intended to guide analytic decisions during the modeling process but rather to aid in the interpretation of findings, particularly in relation to nonsignificant moderation effects. We acknowledge the limitations associated with post hoc power analyses and interpret the results with caution.

We accounted for the potential Type I error inflation across our multiple hypotheses by conducting sensitivity analyses with false discovery rate (FDR) corrections (Benjamini & Hochberg, 1995). The pattern of significance remained consistent, supporting the robustness of the findings for main differences between groups (*t*-tests) and the key effects identified in the regression models, further reinforcing the reliability of the outcomes.

## 4. Results

Table 1 summarizes the sociodemographic characteristics of participants in the research and control groups, including age, ethnicity, education, income, substance use, marital status, parental status, criminal history, and mental health diagnoses.

Regarding the sociodemographic characteristics displayed in Table 1, the age of participants ranged from 18 to 51 in the research group and from 18 to 35 in the control group. The majority of participants (n = 29 in the research group and n = 30 in the control group) self-identified as Latino. Control participants reported higher annual income and educational attainment. Despite their court-mandated probation orders prohibiting alcohol consumption, 33% of participants in this group reported regularly drinking alcohol, and 13.3% reported regularly using illegal drugs. In this group of court-mandated men, 18 participants had been convicted of non-violent crimes in the past, including driving while intoxicated, possession of cannabis, burglary, transportation of undocumented immigrants, illicit drug smuggling, and public intoxication. In addition, 13 participants (9 out of those 18 with non-violent offenses and the other 4 participants) had also committed previous violent crimes different from the IPV incident that put them on probation, including aggravated assault against a male individual, assault on a police officer, assault against a family member different from an intimate partner, and assault on a previous intimate partner. Only one participant in the control group reported having committed a non-violent offense (public intoxication) in the past, and no one reported violent crimes. Eight research participants reported having had mental health problems and having received treatment (medication, psychotherapy, or psychoeducation) for comorbid anxiety and depression (n = 1), depression (n = 3), posttraumatic stress disorder (PTSD, n = 1), PTSD with comorbid anxiety and depression (n = 1), drug use disorder (n = 1), and alcohol use disorder (n = 1). None of the control participants reported having been diagnosed with mental health disorders.

According to the results displayed in Table 1, the control group appeared to have lower socioeconomic status than the control group. Chi-square analyses revealed that differences between both groups were significant for education level χ^2^(3, N = 60) = 9.90, *p* = 0.019 but not for income χ^2^(4, N = 60) = 3.73, *p* = 0.444.

Results for the first hypothesis are shown in Table 2 and Table 3. Table 2 shows the descriptive statistics and the comparison between the variables in the study conducted by independent-samples *t* test, and the two-sided p of their differences between IPV perpetrators (research group) and non-perpetrators (control group).

The outcomes displayed in Table 2 show the significant differences in age between the research and control groups with a large effect size, with perpetrators being, on average, older than non-perpetrators. This result, along with Levene’s test (F = 13.69; sig ≤ 0.001), indicates that equal variances in age cannot be assumed. Yet, significant differences between groups with moderate effect sizes in T and T/C levels, and difficulties in emotional regulation strategies suggest an association of these variables with IPV perpetration or non-perpetration. Notably, the variables awareness and clarity obtained large effect sizes (Cohen’s *d* of 1.246 and 1.077, respectively) and were significant at the 0.001 significance level to differentiate both groups, while the variable impulse obtained a moderate effect size significant at 0.05 level. Perpetrators also had significantly higher total DERS scores than controls. Since we obtained expected cell counts <5 for the nominal variables, we performed Pearson Chi-Square Residual Analysis, and the results are displayed in Table 3.

The adjusted residuals in Table 3 indicate significant differences in ACEs between the control and research groups, since perpetrators of IPV witnessed more IPV and experienced more physical child abuse. The Fisher exact test yielded significant differences at 0.00.

Results for our second hypothesis correspond to the correlations between the variables in the study displayed in Table 4. Regarding group as a variable, correlations have been calculated, accounting for the research group (IPV perpetrators).

Results in Table 4 show negative and significant associations between both T levels and T/C ratios and age, suggesting a drop in these hormones associated with aging. Being in the research group was positively and significantly associated with T, T/C, having witnessed IPV, having been physically abused in childhood, and difficulties in emotion regulation (impulse, awareness, clarity, and general difficulties in emotion regulation—DERS). Additionally, some of these difficulties in emotional regulation were apparently positively associated with age, which is consistent with the higher mean age in the research group. Whereas, higher education levels were negatively correlated with T levels, TC ratios, impulsivity, and lack of awareness. Results in this table also indicate that witnessing IPV was weakly and positively correlated with impulsivity, lack of awareness, and clarity. Similarly, child abuse was positively correlated with lack of awareness and clarity, and physical abuse from parents was positively correlated with lack of awareness and clarity. Since lack of emotional awareness met the two conditions: being a significant variable to differentiate the subjects in the control group from those in the research group and showing significant correlations with the predictor variables (T levels and T/C ratios), regression analyses have been conducted to test our third hypothesis by testing this variable as a moderator between T and T/C and the probability of being in the control (non-perpetrator) or the research (perpetrator) group. In the case of the variable awareness the estimated statistical power was 1.0, suggesting that our sample was adequately powered to detect an effect. Age was introduced as a covariate, given that it was correlated with T levels and T/C ratios and equal variances cannot be assumed for this variable in the two compared groups and there were significant age differences between the two compared groups. These results are shown in Table 5 and Table 6. Given that education level significantly correlated with both T levels and T/C ratios, it was initially tested as a covariate in the regression models. However, its inclusion did not significantly alter the results and was therefore excluded from the final models for the sake of parsimony.

Results in Table 5 suggest age accounts for almost 28% of the variance (Nagelkerke R^2^ = 0.279). T, when added to the model, is confirmed to be a significant predictor (<0.001), as its inclusion increases the prediction of the regression model to account for almost 69% of the variance (Nagelkerke R^2^ = 0.688), as well as lack of emotional awareness, which increases the prediction up to almost 76% (Nagelkerke R^2^ = 0.759, sig = 0.018), while the interaction between these two last variables barely increases predictability up to 79% (Nagelkerke R^2^ = 0.792 sig ≤ 0.001). However, the analysis of the interaction between emotional awareness and testosterone did not confirm lack of emotional awareness as a significant moderator (sig. = 0.081), even when all the variables, including lack of emotional awareness (by its own) remained significant at 0.05 level on step 4. Nevertheless, the Nagerlkerke R^2^ increased from 0.759 to 0.792, suggesting that the moderation increased the prediction of the model. Regression analysis yielded an overall percentage of accurately predicting 56 cases (control group = 93.3%; research group = 93.3%).

Similar results to those in Table 5 were obtained when introducing T/C instead of T as shown in Table 6. T/C ratio is a stronger predictor than T levels and the total model accounts for more variance, up to 98% (Nagelkerke R^2^ = 0.798, sig ≤ 0.001), than that with T levels. Regression analysis yielded an overall percentage of accurately predicting 55 cases (control group = 90%; research group = 93.3%). Although all the variables, including lack of emotional awareness (by its own) remained significant at 0.05 level on step 4, the analysis did not confirm lack of awareness as a significant moderator (sig. = 0.183) interacting with T/C ratios in the sample.

Results in Table 7 show logistic regressions performed to test our fourth hypothesis by introducing age as a covariate and having witnessed IPV between parents in childhood, T levels, and lack of emotional awareness (model one), lack of emotional clarity (model two) and impulse (model three) as independent variables predicting for group. Logistic regression model one was statistically significant, χ^2^(4) = 64.161, *p* < 0.001and explained 87.6% (Nagelkerke R^2^) of the variance in IPV perpetration and correctly predicted 91.7% of the cases (research = 90%; control = 93.3%). Logistic regression model two was also statistically significant, χ^2^(4) = 60.579, *p* < 0.001, explained 84.8% (Nagelkerke R^2^) of the variance in IPV perpetration and correctly predicted 91.7% of the cases (research = 86.7%; control = 96.7%). Even when difficulties in impulse control was not a significant variable in model three, the model was statistically significant χ^2^(4) = 55.869, *p* < 0.001, explained 80.8% (Nagelkerke R^2^) of the variance and correctly predicted 88.3% of the cases (research = 86.7%; control = 90%).

Models four, five and six (also shown in Table 7) included age as a covariate and having witnessed IPV between parents in childhood, T/C ratios and lack of emotional awareness (model four), lack of emotional clarity (model five) and difficulties in impulse control (model six), as independent variables. Logistic regression model four was statistically significant, χ^2^(4) = 62.696, *p* < 0.001, explained 86.4% (Nagelkerke R^2^) of the variance in IPV perpetration, and correctly predicted 93.3% of the cases (research = 90%; control = 96.7%). Logistic regression model five was statistically significant, χ^2^(4) = 69.211, *p* < 0.001, explained 91.3% (Nagelkerke R^2^) of the variance in the outcome variable, and correctly predicted 96.7% of the cases (research = 96.7; control = 96.7%). Model six was also statistically significant χ^2^(4) = 58.411, *p* < 0.001, and even when impulse was not a significant factor, its addition to the model increased its ability to explain variance up to 82.2%, and the model correctly predicted 90% of the cases (research = 90; control = 90%).

Binary logistic regression utilizing age as a covariate, yielded significant outcomes for antecedents of physical child abuse (sig ≤ 0.001; Nagelkerke R^2^ = 0.530), T levels (sig = 0.007; Nagelkerke R^2^ = 0.871), but not for any of the difficulties in emotion regulation variables. When introducing T/C ratios instead of T levels in the regression model, similar results were obtained (T/C sign = 0.005; Nagelkerke R^2^ = 0.881).

## 5. Discussion

As predicted, perpetrators of IPV exhibited greater deficits in emotional regulation and reported having witnessed more interparental violence and more frequently been victims of child abuse than non-aggressive controls. With respect to emotional regulation, they reported more emotional regulation deficits in general and specifically more difficulties with emotional awareness, clarity and impulsivity. These difficulties in emotional regulation were found to be positively related to both testosterone and T/C levels as well as to having witnessed and experienced child abuse. More specifically, having experienced child abuse was associated with greater overall emotional regulation difficulties and specifically, greater difficulties with emotional awareness and clarity and having witnessed IPV between one’s parents in childhood was associated with impulsivity, emotional awareness and clarity. As reported in (Cantos et al., 2024) there were negative and significant associations between both T levels and T/C ratios and age, indicating a drop in these hormones was associated with aging. The only emotional regulation variable to be related to both testosterone and the testosterone cortisol ratio and perpetration of IPV was lack of emotional awareness. However, while lack of emotional awareness independently predicted IPV perpetration, it did not moderate the relationship between testosterone and perpetration which suggests that both greater testosterone and difficulties with emotional awareness independently influence the perpetration of intimate partner violence. Thus, elevated levels of testosterone may impair people’s ability to be aware of their emotions which in turn may increase the probability of perpetration of IPV, but a person’s lack of emotional awareness does not appear to influence whether someone with high levels of testosterone will perpetrate violence against an intimate partner. It appears that testosterone levels, having experienced child abuse and witnessed interpersonal violence in childhood, all three of which independently predict IPV perpetration, may also all be likely to predispose an individual towards difficulties with emotional regulation which in turn may also increase the likelihood of perpetration of intimate partner violence,

Models including the conjunction of T levels and T/C ratios, as biological variables and ACEs (child abuse, witnessing IPV) as environmental variables, and emotional regulation difficulties, as psychological variables, were found to adequately predict the probability of IPV perpetration, suggesting that we need to take biological, environmental and psychological variables into consideration in understanding the occurrence of IPV and in developing interventions to reduce the likelihood of its occurrence and provides support for biopsychosocial multicausal approaches to understanding and addressing it.

These results are consistent with studies identifying having been abused as a child and witnessing IPV between parents as risk factors for IPV perpetration in adulthood (Black et al., 2010; Ehrensaft & Langhinrichsen-Rohling, 2022; Islam et al., 2017; Manchikanti-Gómez, 2011; Puno et al., 2023). Results are also consistent with studies showing a relationship between having been abused and having witnessed interparental violence in childhood and emotional dysregulation (Meddeb et al., 2023; Oliveros & Coleman, 2019; Orozco-Vargas et al., 2021). Of particular interest is Zhang et al.’s (2023) review that determined early exposure to IPV to negatively affect the development of self-regulatory strategies, including emotional and behavioral regulation.

**Figure 1:**
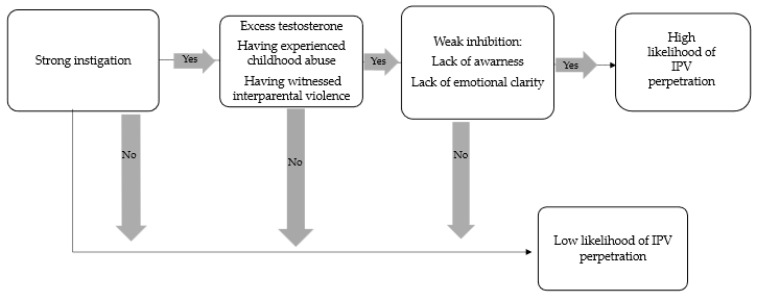
Influence of psychosocial variables and biomarkers on IPV risk based on the I^3^ and perfect storm frameworks.

The results support I^3^ theory (Finkel, 2014) which posits three factors, instigation, impellance and inhibition, that influence the likelihood and intensity of a given behavior, including aggression. Excess testosterone, having experienced childhood abuse, having witnessed interparental violence and emotional regulation difficulties would all be impellance factors defined as situational or dispositional qualities that increase the likelihood or intensity of a person’s procilivity to enact IPV when encountering an instigating trigger. Perfect storm theory (Finkel & Eckhardt, 2013) which is derived from the I^3^ model further suggests that a behavior is most likely to occur when instigation and impellance are strong and inhibition is weak. These results would suggest that IPV perpetrators have strong impellance factors and weak inhibitory factors which increases the likelihood of perpetration when they encounter an instigator factor such as a conflict situation with their partner. Figure 1 provides a conceptual representation of how key constructs from the I^3^ and perfect storm models map onto the results.

### 5.1. Clinical Implications

As mentioned above, these findings have implications for interventions to reduce the frequency of IPV perpetration in adulthood. The deleterious wide-ranging effects of having been abused as a child and exposure to interparental violence are further reinforced by the results of this study. It is important and crucial that primary prevention efforts to reduce the likelihood of these events occurring continue to be given priority by society as a whole. The importance of continuing to educate the public as to their detrimental effects should be a priority for all those involved in public health policies. The identification of those at high risk for perpetration and providing these populations with coping strategies to reduce the risk is likely to have a positive impact for society, both in the short and long term. Providing expectant mothers and fathers with specific education on the occurrence of child abuse and providing them with coping strategies to deal with the stressors of parenthood might have a positive impact. Additionally, providing adolescents with information on the occurrence of partner violence as well as providing them with training in emotional awareness and relationship skills such as positive communication and conflict resolution strategies will also likely have an impact on reducing the frequency of IPV in adulthood. With respect to secondary prevention, first and foremost, the need for prevention interventions with children who have been victims of child abuse and have witnessed interparental violence is apparent. These interventions need to be focused on understanding the impact of both experiencing child abuse and witnessing interparental violence as well as providing these children with constructive self-regulatory strategies including training in emotional awareness, and general emotional regulation and behavioral regulation skills. Providing these children with non-violent conflict resolution and communication skills is also important in light of the negative models they have been exposed to. Finally, providing exposure to examples of healthier non-violent relationships may also help ameliorate the adverse impact of the violent relationships they have been exposed to while providing models that they may emulate and thus reduce the risk of perpetration of IPV in adulthood.

Interventions with adults that have already perpetrated violence against their partners need to assess for the presence or absence of having been abused and having witnessed interparental violence in childhood, as well as for emotional regulation and communication difficulties. It is important to allow perpetrators who have experienced child abuse and witnessed interparental violence to discuss these experiences as well as the impact they have had on those that have experienced it. Zarling et al. (2025) compared acceptance and commitment therapy (ACT) to a Duluth/Cognitive Behavioral Intervention (DCBT) and they found that ACT men had fewer DV charges as well as fewer any violence (non-IPV) charges. In examining mechanisms of change, Zarling et al. (2025) found that psychological flexibility, an ability to remain present and open to experiences even though the thoughts and emotions are stressful, was associated with reductions in IPV. Given the extensive research showing that exposure to IPV and being the victim of abuse from a parent are both risk factors for engaging in IPV, it seems quite logical for therapists treating men who have engaged in IPV to explore their own family violence history and to discuss how to prevent an intergenerational cycle of violence. Indeed, we have advocated for a discussion of the cycle of violence and lessons learned from family of origin violence for years, and our treatments which incorporated such had significant reductions in IPV (O’Leary et al., 1999). Finally, as suggested by Cantos et al. (2024) it might be beneficial to provide relapse prevention types of interventions which would train perpetrators in responding to high-risk situations and providing those high on testosterone with information regarding their propensity to react to certain types of situations in an aggressive and impulsive manner. This may reduce the likelihood that they respond aggressively to conflictual situations with their partner. However, this suggestion is speculative and is suggested as a future avenue for empirical investigation. There is no evidence that informing men that they have high T levels that could make them at higher risk for IPV would help them desist from engaging in IPV, and more replications and extensions of research on the T/IPV link are needed before any prevention strategy of an information kind seems warranted. The link between T and IPV may be sample dependent as suggested by Archer (2006) in his meta-analysis with men with criminal and/or highly aggressive histories being the group in which the link between T and IPV is most likely evident. It is important that these interventions not be based on a one size fits all approach (Cantos & O’Leary, 2014) and instead be based on careful and comprehensive assessments of perpetrators on relevant dimensions that have been identified empirically as implicated in the perpetration of intimate partner violence.

### 5.2. Limitations

Results are based on a small sample size (n = 60) from a border city in Texas which has a majority Hispanic population so the results may not be generalizable to the rest of the population of men who aggress against their partner. Conversely, it contributes to an understanding of the characteristics of Hispanic (mostly of Mexican American origin) perpetrators of intimate partner violence on probation, in a specific region of the US. These results are based on men mandated to court ordered programs and the results may not generalize to men who are not court mandated. Of the men mandated to court ordered program herein, 18 of the 60 had been convicted of non-violent crimes, and 13 of the 60 had committed non-violent crimes other than the IPV incident that led to their arrest. In short, a significant percentage of these men had legal problems beyond their IPV problems. Relatedly, in a meta-analysis the association of baseline T and aggression was strongest among offenders, a group of individuals with elevated levels of impulsivity (Geniole et al., 2020). Further, early studies of experimentally manipulated T levels in men have found mixed results regarding the association of T with aggression (O’Connor et al., 2002).

There was a significant age difference between the perpetrators and the non-violent controls. The average age of the perpetrators was 30.80 and the average age of the controls was 24.13. Consequently, age was used as a covariate in the analyses. There is convincing epidemiological data to show that serum free and total testosterone fall with normal aging (Feldman et al., 2002). Further, one would assume that testosterone levels in men in their 30 s would be slightly lower than the testosterone levels of men in their 20 s. The observed testosterone differences between the perpetrators of IPV (413.89) and non-violent controls (333.99) are in accord with our predictions, but younger men would be expected to have higher T values than older men. Thus, age was used as a covariate to control for this difference statistically. It is recommended that future studies attempt to match the different groups based on age to further test this difference.

Given the small sample size, the inclusion of multiple predictors and interaction variables in the logistic regression models may increase the risk of overfitting. While the results provide suggestive evidence in support of our hypotheses, they should be interpreted with caution and validated in larger samples.

Lastly, it is important to remind the reader that this is a correlational study and that while the reported directionality is supported by the literature, the study’s correlational design cannot establish causality.

## Figures and Tables

**Table 1:** Sociodemographic characteristics of the research and control groups.

Variable	Research Group (n = 30)	Control Group (n = 30)
Age (years)	M = 30.80, SD = 8.43	M = 24.13, SD = 4.28
Ethnicity		
Latino	83.3% (n = 29)	100% (n = 30)
African American	3.3% (n = 1)	0% (n = 0)
Annual Income		
<$21,000	50.0% (n = 15)	66.6% (n = 20)
$21,000–$45,000	50.0% (n = 15)	33.3% (n = 10)
Education Level		
<4th grade	10.0% (n = 3)	3.3% (n = 1)
High school	63.3% (n = 19)	46.6% (n = 14)
Associate degree	20.0% (n = 6)	10.0% (n = 3)
Bachelor’s degree	6.6% (n = 2)	40.0% (n = 12)
Substance Use		
Regular alcohol use	33.3% (n = 10)	56.7% (n = 17)
Regular illegal drugs use	13.3% (n = 4)	20.0% (n = 6)
Marital Status		
Single	40.0% (n = 12)	70.0% (n = 21)
Married	10.0% (n = 3)	10.0% (n = 3)
Divorced	13.3% (n = 4)	3.3% (n = 1)
Living with partner	36.6% (n = 11)	16.6% (n = 5)
Parental Status		
Has children	83.3% (n = 25)	10.0% (n = 3)
Number of children	*M* = 2.33 (min = 1, max = 6)	*M* = 1.33 (min = 1, max = 2)
Criminal History		
Non-violent offenses	60.0% (n = 18)	3.3% (n = 1)
Violent offenses	43.3% (n = 13)	0.0% (n = 0)
Mental Health Diagnosis	26.6% (n = 8)	0.0% (n = 0)

Note. Values are presented as percentages with raw counts in parentheses. Percentages may not total 100 due to rounding. Some participants report multiple categories (e.g., criminal history and mental health).

**Table 2:** Comparisons Between Perpetrators and Non-Perpetrators in Age, T, T/C, and Difficulties in Emotion Regulation.

	Research (n = 30)		Control (n = 30)		*t*	*p*	Cohen’s *d*	95% CI	
	*M*	*SD*	*M*	*SD*				LL	UL
Age	30.80	8.43	24.13	4.28	3.86	<0.001	0.997	0.456	1.531
T	413.89	126.11	333.99	110.73	2.61	0.012	0.673	0.150	1.191
T/C	0.064	0.020	0.051	0.017	2.55	0.014	0.658	0.136	1.176
Acceptance	12.0	5.24	13.06	5.86	−7.42	0.461	−0.192	−0.698	0.316
Goal	12.53	5.27	11.83	4.24	5.67	0.573	0.146	−0.361	0.652
Impulse	12.53	5.44	9.70	3.57	2.38	0.021	0.615	0.095	1.131
Awareness	19.93	5.09	14.00	4.40	4.83	<0.001	1.246	0.687	1.795
Strategies	14.87	5.30	13.10	4.37	1.41	0.164	0.364	−0.148	0.873
Clarity	13.73	4.01	9.63	3.60	4.17	<0.001	1.077	0.530	1.615
DERS	85.60	23.12	71.33	16.95	2.73	0.009	0.704	0.179	1.223

Note. Confidence intervals (CI), lower limit (LL), upper limit (UL).

**Table 3:** ACEs in Control and Research Group. Crosstabulation.

		Child Abuse (No)	Child Abuse (Yes)	Witnessed IPV (No)	Witnessed IPV (Yes)
Control (n = 30)	Count	25	5	27	3
	Expected Count	16.5	13.5	19.5	10.5
	Percentage	83.3%	16.7%	90%	10%
	Adjusted Residual	4.4 **	−4.4 **	4.1 **	−4.1 **
Research (n = 30)	Count	8	22	12	18
	Expected Count	16.5	13.5	19.5	10.5
	Percentage	26.7%	73.3%	40%	60%
	Adjusted Residual	−4.4 **	4.4 **	−4.1 **	4.1 **
Total (N = 60)	Count	33	27	39	21
	Expected Count	33	27	39	21.0
	Percentage	55%	45%	65%	35%

Note. ** significant at the 0.00 (2-tailed).

**Table 4:** Bivariate Correlations Between the Variables in the Study.

	Age	T	T/C	Wit	Group	Acc	G	Imp	Aw	St	Cl	DERS	Inc	ChA
Age	-	−0.443 **	−0.446 **	-	0.452 **	0.171	0.336 **	0.294 *	0.233	0.266 *	0.247	0.357 *	-	-
T	-	-		-	-	−0.283 *	−0.208	0.081	0.315 *	−0.024	0.219	0.020	-	-
T/C	-	0.981 **	-	-	-	−0.298 *	−0.231	0.054	0.287 *	−0.065	0.179	−0.020	-	-
Wit	0.233	0.096	0.060	-	-	−0.141	0.127	0.262 *	0.307 *	0.168	0.316 *	0.233	-	-
ChA	0.293 *	−0.027	−0.053	0.601 **	0.570 **	−0.015	0.043	0.225	0.392 **	0.176	0.342 **	0.268 *	-	-
Group	-	0.324 *	0.317 *	0.524 **	-	−0.097	0.074	0.299 *	0.535 **	0.182	0.480 **	0.337 **	-	-
EL	0.102	−0.383 **	−0.348 **	−0.211	−0.344 **	0.113	−0.104	−0.301 *	−0.349 **	−0.173	−0.070	−0.206	0.209	−0.213
ChA	0.293 *	−0.027	−0.053	0.601 **	0.570 **	−0.015	0.043	0.225	0.392 **	0.176	0.342 **	0.268 *	−0.231	
Inc	0.226	0.014	0.046	−0.289*	0.187	−0.005	−0.043	−0.090	0.000	0.016	0.008	−0.026	-	-

Note. N = 60, ** Correlation is significant at the 0.01 level (2-tailed); * Correlation is significant at the 0.05 level (2-tailed). Wit (Witnessing IPV), ChA (child abuse), education level (EL), child abuse perpetrated by parents (ChA), annual income (Inc), Acceptance (Acc), goal (G), impulse (Imp), awareness (Aw), strategies (St), clarity (Cl).

**Table 5:** IPV Perpetration Predicted from T Levels and Moderated by Lack of Emotional Awareness with Covariate for Age.

Predictor	Step 1	Step 2	Step 3	Step 4	Nagelkerke R Square Change
Age					0.279
*B*	0.164 *	00.407 **	0.445 **	0.604 *	
*SE*	0.053	0.106	0.135	0.198	
T levels					0.688
*B*	-	0.020 **	0.019 **	0.023 *	
*SE*	-	0.005	0.006	0.007	
Awareness	-				0.759
*B*	-	-	0.273 *	0.356 *	
*SE*	-	-	0.116	0.152	
T × Awareness					0.792
*B*	-	-	-	−1.472	
*SE*	-	-	-	0.845	

Note. Unstandardized regression coefficients (*B*), standard error (*SE*), * *p* ≤ 0.05., ** *p* ≤ 0.01.

**Table 6:** IPV Perpetration Predicted from T/C Ratios and Moderated by Lack of Emotional Awareness with Covariate for Age.

Predictor	Step 1	Step 2	Step 3	Step 4	Nagelkerke R Square Change
Age					0.279
*B*	0.164 *	0.42 **	0.510 *	0.601 *	
*SE*	0.053	0.109	0.161	0.190	
T/C					0.691
*B*	-	129.40 **	139.08 **	150.15 *	
*SE*	-	33.69	40.95	45.93	
Awareness	-				0.780
*B*	-	-	0.337 *	0.392 *	
*SE*	-	-	0.134	0.162	
T/C × Awareness	-	-	-		0.798
*B*	-	-	-	−1.11	
*SE*	-	-	-	0.834	

Note. * *p* ≤ 0.05., ** *p* ≤ 0.01.

**Table 7:** Binary Logistic Regressions. Group predicted by ACE’s, T levels, T/C Ratios and Difficulties in Emotion Regulation.

Variable	*B*	*SE*	Wald χ^2^	Sig.	Odds	Nag. R^2^
1. Age	0.882	0.352	6.263	0.012	2.415	0.279
Witnessing IPV	6.044	2.425	6.212	0.013	421.670	0.514
T levels	0.033	0.012	6.994	0.008	1.033	0.793
Awareness	0.474	0.206	5.273	0.022	1.606	0.876
2. Age	0.669	0.236	8.072	0.004	1.953	0.279
Witnessing IPV	4.498	1.739	6.693	0.010	88.819	0.514
T levels	0.025	0.008	8.741	0.003	1.025	0.793
Clarity	0.411	0.199	4.257	0.039	1.509	0.848
3. Age	0.570	0.192	8.829	0.003	1.769	0.279
Witnessing IPV	3.987	1.514	6.936	0.008	0.008	0.514
T levels	0.022	0.007	9.333	0.002	0.002	0.793
Impulse	0.200	0.160	1.570	0.210	0.210	0.808
4. Age	0.734	0.269	7.455	0.006	2.084	0.279
Witnessing IPV	4.998	1.986	6.335	0.012	148.144	0.514
T/C ratios	169.815	57.905	8.600	00.003	5.620 × 10^73^	0.804
Clarity	0.487	0.234	4.321	0.038	1.628	0.864
5. Age	1.224	0.503	5.929	0.015	3.402	0.279
Witnessing IPV	8.232	3.372	5.958	0.015	3759.097	0.514
T/C ratios	294.606	118.592	6.171	0.013	8.822 × 10^127^	0.804
Awareness	0.692	0.294	5.549	0.018	1.998	0.913
6. Age	0.666	0.236	7.938	0.005	1.946	0.279
Witnessing IPV	4.641	1.777	6.824	0.009	103.648	0.514
T/C ratios	160.990	53.671	8.998	0.003	8.266 × 10^69^	0.793
Impulse	0.286	0.176	2.627	0.105	1.331	0.822

## Data Availability

The data presented in this study are not available due to privacy since the research group was on probation for IPV.
